# Deep Learning on High-Throughput Transcriptomics to Predict Drug-Induced Liver Injury

**DOI:** 10.3389/fbioe.2020.562677

**Published:** 2020-11-27

**Authors:** Ting Li, Weida Tong, Ruth Roberts, Zhichao Liu, Shraddha Thakkar

**Affiliations:** ^1^Division of Bioinformatics and Biostatistics, National Center for Toxicological Research, U.S. Food and Drug Administration, Jefferson, AR, United States; ^2^Joint Bioinformatics Program, University of Arkansas at Little Rock and University of Arkansas for Medical Sciences, Little Rock, AR, United States; ^3^ApconiX Ltd., Alderley Edge, United Kingdom; ^4^Department of Biosciences, University of Birmingham, Birmingham, United Kingdom

**Keywords:** DILI, deep learning–artificial neural network, high throughput transcriptomics, toxicity prediction model, machine learning, risk assessment

## Abstract

Drug-induced liver injury (DILI) is one of the most cited reasons for the high drug attrition rate and drug withdrawal from the market. The accumulated large amount of high throughput transcriptomic profiles and advances in deep learning provide an unprecedented opportunity to improve the suboptimal performance of DILI prediction. In this study, we developed an eight-layer Deep Neural Network (DNN) model for DILI prediction using transcriptomic profiles of human cell lines (LINCS L1000 dataset) with the current largest binary DILI annotation data [i.e., DILI severity and toxicity (DILIst)]. The developed models were evaluated by Monte Carlo cross-validation (MCCV), permutation test, and an independent validation (IV) set. The developed DNN model achieved the area under the receiver operating characteristic curve (AUC) of 0.802 and 0.798, and balanced accuracy of 0.741 and 0.721 for training and an IV set, respectively, outperforming the conventional machine learning algorithms, including *K*-nearest neighbors (KNN), Support Vector Machine (SVM), and Random Forest (RF). Moreover, the developed DNN model provided a more balanced sensitivity of 0.839 and specificity of 0.603. Besides, we found the developed DNN model had a superior predictive performance for oncology drugs. Also, the functional and network analysis of genes driving the predictions revealed their relevance to the underlying mechanisms of DILI. The proposed DNN model could be a promising tool for early detection of DILI potential in the pre-clinical setting.

## Introduction

Drug-induced liver injury (DILI) has been recognized as a significant cause of drug attrition, resulting in drug withdrawal from any stage of the drug development processes and post-marketing (Hoofnagle and Björnsson, 2019; Weaver et al., 2020). DILI accounts for approximately 50% of acute liver failure cases in the United States (Ostapowicz et al., 2002). Notably, DILI risk covers more than 750 approved drugs (Thakkar et al., 2018). The late stage of DILI identification poses a serious challenge to pharmaceutical professionals as well as regulatory agencies. Therefore, early elimination of DILI concerns in the pre-clinical setting is of great importance for DILI management (Weaver et al., 2020).

Drug-induced liver injury is a very composite endpoint in predictive toxicology, which interplays between drug properties and host factors (Chen et al., 2015). Besides the clinical-driven approaches initialized by consortiums such as DILI Network (DILIN) (Fontana et al., 2009), considerable efforts have been made for enhancing DILI prediction in a pre-clinical setting (Walker et al., 2020). Poor extrapolation from pre-clinical animal models to clinical hepatotoxicity exists. Thus, the paradigm has been shifted to developing *in vitro* (Chan and Benet, 2018; Tolosa et al., 2018; Aleo et al., 2020) and *in silico* (Mulliner et al., 2016; Hong et al., 2017; Shin et al., 2020) models for DILI prediction. Notably, toxicogenomics (TGx), integrating genomics and computational approaches, becomes a new trend to enhance DILI prediction (Liu et al., 2019). Wang et al. (2019) developed Deep neural networks (DNNs) by using rat transcriptomic profiles from the Open TG-GATEs database (Igarashi et al., 2014) to predict three DILI endpoints including biliary hyperplasia, fibrosis, and necrosis. The model yielded the Matthews correlation coefficients (MCC) of 0.56∼0.89. Although it is still elusive of the model performance in human DILI endpoints, it is a beneficial attempt. Kohonen et al. (2017) developed a “predictive toxicogenomics space (PTGS)” tool composed of 1, 331 genes based on Connectivity Map (CMap) and NCI-60 *in vitro* assays. The developed PTGS tool was applied for human DILI prediction with different DILI annotation datasets and achieved a sensitivity of 72–86% without a loss of specificity.

It is challenging to evaluate and cross-compare the performance metrices such as sensitivity and specificity of the reported DILI models directly. First, there is a lack of a consistent DILI classification scheme to standardize and unify the existing DILI annotation strategies (Walker et al., 2020). In the past decade, the FDA National Center for Toxicological Research (NCTR) research team continue to minimize the discrepancies among the different reported DILI classifications. Such efforts were incorporated into the FDA’s Liver Toxicity Knowledge Base (LTKB) with two developed DILI classification datasets - LTKB Benchmark Dataset (Chen et al., 2011) and DILIrank (Chen et al., 2016). Recently, the FDA team released DILI severity and toxicity (DILIst) dataset (Thakkar et al., 2020). The DILIst provides access to the largest binary DILI classification dataset composed of 1279 drugs, accurately categorized for DILI potential by the assimilation of highly concordant information on DILI from clinical evidence, literature evidence, case registry and the FDA Adverse Event Reporting System (FAERS). Second, the sample size varies among the reported model is another hurdle to compare their model performance. Furthermore, the number of samples used in the published models is usually very limited. For example, the large-scale of TGx, such as the Open TG-GATEs database consists of 170 compounds with four different types of assays, i.e., rat/human *in vitro*, and rat *in vivo* single/repeated doses. However, the small number of DILI negative compounds limits its application for predictive DILI model development. Benefited from high-throughput screen technologies such as L1000 (Subramanian et al., 2017) and TempO-Seq (Bushel et al., 2018), transcriptomic profiles for millions of compounds designed to monitor hundreds to thousands of genes could be generated at once in high throughput at an incredibly lower cost. Accumulated transcriptomic profiles generated from these technologies could potentially improve the generalization of the DILI model. Last, there is a lack of a comprehensive assessment of machine learning (ML) algorithms for their DILI predictive ability. So far, there have been limited investigations that evaluated the superiority of deep learning (DL) over conventional ML in the context of toxicity prediction. Some observations were made with only small datasets and were based just on chemical structure data (Muratov et al., 2020). Further evaluation of DL algorithms in diverse biomedical profiles is urgently needed for better understating of its potential in DILI prediction.

In this study, we developed a DNN model for DILI prediction based on the largest binary DILI classification dataset–DILIst as well as the transcriptomic profiles from the Library of Integrated Network-based Cellular Signatures (LINCS) L1000 dataset. To our best knowledge, it is the first attempt in the community. The developed DNN models were comprehensively evaluated by using Monte Carlo cross-validation (MCCV), a permutation test, and an independent validation (IV) set. The performance of the developed DNN models was also intensively compared to the conventional ML algorithms, including *K*-nearest neighbors (KNN), Support Vector Machine (SVM), and Random Forest (RF). Moreover, the applicability domain and biological relevance of the proposed DNN model were also defined and investigated through various approaches such as therapeutic class analysis, pathway analysis, and network analysis.

## Materials and Methods

### Data Preparation

#### Drug-Induced Liver Injury (DILI) Annotation Data

Drug-induced liver injury severity and toxicity developed by the FDA’s NCTR was employed, which is the largest binary DILI annotation dataset (Thakkar et al., 2020). DILIst integrated the human hepatotoxicity-related evidence derived from approved drug labeling, hepatotoxic case registries, FAERS, and literature reports. The current version of DILIst contains a total of 1279 drugs, where 768 are DILI positives, and 511 are DILI negatives.

#### High-Throughput Transcriptomic Profiles

Drug-induced transcriptome profiles used for this study were curated from the NIH LINCS L1000 dataset (GEO accession number: GSE92742) (Subramanian et al., 2017). LINCS L1000 dataset consists of over 1.3 million transcriptomic profiles across human cell lines. The data generated from LINCS L1000 has been subjected to a data processing pipeline established by the LINCS consortium and to produce data at five levels. In this study, we used Level 5 moderated *Z*-scores data that contained 473,647 collapsed transcriptomic profiles derived from the weighted averages of the individual replicates on 978 landmark genes. Here, each transcriptomic profile of Level 5 represents the treatment effect of a drug/dose/duration/cell line combination.

#### Transcriptomic Profiles for Model Development

We mapped LINCS L1000 Level 5 data onto the DILIst based on their shared drug names and related synonyms through PubChem Identifier Exchange Service^1^. As a result, we obtained 23,791 transcriptomic profiles across 69 human cell lines. Here, we hypothesized the DILI signal was embedded in transcriptomic profiles with different drug/dose/duration/cell line combinations. However, based on our previous study, the correlation between liver-related cell lines and various cancer cell lines varied (Liu et al., 2020). Therefore, we used a modified Kennard-Stone algorithm (Kennard and Stone, 1969) for sample selection to extract transcriptomic profiles with the most explained variance. Accordingly, we calculated a average transcriptomic profile based on all the 23,791 transcriptomic profiles. Then, we calculated Euclidean distances between average transcriptomic profile and each of 23,791 transcriptomic profiles. Transcriptomic profiles with Euclidean distances located in two sides of the quantile value 0.05 (e.g., a confidence interval 0.95) were selected (see the Supplementary Figure S1). Consequently, 6,000 transcriptomic profiles were obtained for model development, of which 3,568 were DILI positives and 2,432 were DILI negatives (e.g., positive/negative ratio = 1.47). The detailed information, including drug name, concentration, cell line, and DILIst label, was listed in the Supplementary Table S1.

### Description of Classifiers

The study aims to develop a DNN model based on transcriptomic profiles for DILI prediction. Furthermore, we compared the DNN model performance with three state-of-the-art ML algorithms, including KNN, SVM, and RF.

#### Deep Neural Network

A DNN is an artificial neural network (ANN) consisting of multiple layers between the input and output layers (Schmidhuber, 2015). The DNN aims to find the correct mathematical manipulation to transform the input into the output through a linear or a non-linear relationship. The network moves through the layers by calculating the probability of each output. In this study, we developed a DNN model with seven hidden layers between the input and output layers (Supplementary Figure S2). The nodes in the input layer were loaded with the feature information of transcriptomic profiles. Then, the input layer information was forwarded through the network of seven hidden layers and then finally to the output layer (i.e., probability of DILI classification). For each node in the hidden layers, and the output layer, the weighted sum (${\text{S}}_{j}^{l}$) was calculated using the formula below:

(1) Sjl=∑k⁢Wj⁢kxkl*+l-1bjl

Where $x_{k}^{l-1}$ is the *k*th node in the layer of *l-1*, $W_{j\,k}^{l}$ is the weight between $x_{k}^{l-1}$ and $x_{j}^{l}$, $b_{j}^{l}$ is on the bias for neuron $x_{j}^{l}$. Then the weighted sum was activated through a non-linear function as shown in the following formula:

(2) $$x_{k}^{l}=f\,\left({\text{S}}_{j}^{l}\right)$$

Here, $x_{k}^{l}$ denotes the output of an activation function. The activation for the hidden layers was optimized during the model construction. In this study, we investigated four activation functions, including Hyperbolic tangent activation (Tanh), Rectified Linear Unit (ReLU), Scaled Exponential Linear Unit (SELU), and Exponential Linear Unit (ELU). For the output layer, we used the sigmoid activation function for DILI classification prediction (binary where DILI negative prediction = 0 and DILI positive predictions = 1) (if the sigmoid output value ≤ 0.5, classify to DILI negative, else classify to DILI positive). The binary cross-entropy loss function was applied to calculate the loss of the predicted value $\hat{Y_{l}}$ and the actual value _*Y*_*i*__. The loss function was presented by the following formula:

(3) $$L\,\left(Y_{i},\hat{Y_{l}}\right)=-\sum_{1}^{n}\left(Y_{i}\,\left(\log\left(\hat{Y_{l}}\right)\right)+\left(1-Y_{i}\right)\,\log\left(1-\hat{Y_{l}}\right)\right)$$

Four optimizers (i.e., Adam, Adadelta, RMSProp, and SGD) were employed and applied to minimize the loss function by backward updating the weights and bias on the DNN training process.

#### KNN

*K*-nearest neighbors is a non-parametric algorithm that determines each test object class label as the most frequent one among the *k*-nearest training objects (Altman, 1992). In this study, we used Euclidean distance to measure the distance between the test object and the training object. The hyperparameter (the number of neighbors *K*) was optimized.

#### SVM

Support vector machine is a supervised ML algorithm developed by Cortes and Vapnik (1995). It separates objects by constructing a hyperplane based on support vectors. In addition to performing linear classification, SVM can also be used to perform non-linear classification using kernel functions. The kernel function can project the original feature space into a higher dimensional feature space, which may help to separate objects into different classes. In this study, we tested polynomial, and radial basis function (RBF) kernel functions with hyperparameters, i.e., Gamma for the non-linear kernel, and penalty parameter C.

#### RF

Random forest is an ensemble learning model used for classification by building multiple decision trees, and the output of the decision is the ensemble result of all the individual trees. The hyperparameters, including the number of trees, the max depth of the tree, minimum sample split, and minimum sample leaf, were optimized.

### Model Development

**FIGURE 1:**
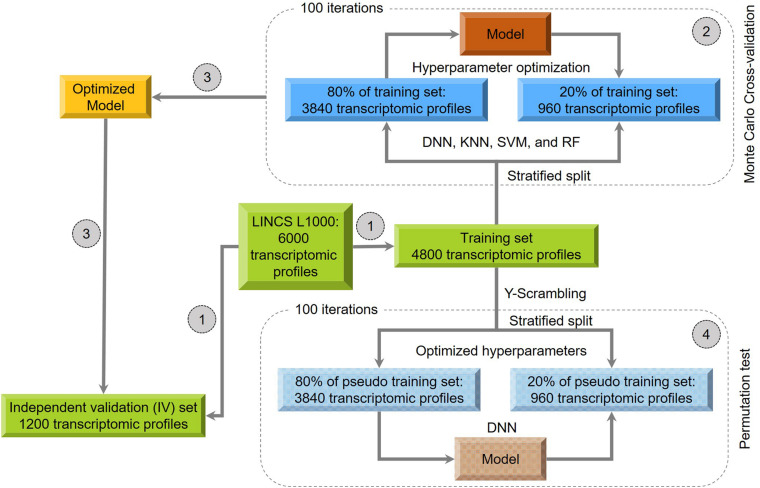
Overall workflow for the model development: (1) 6000 treatments were stratified split into the training (80%) and independent validation (20%) datasets. (2) A 100-iteration Monte Carlo cross-validation (MCCV) was carried out for hyperparameter optimization of the four algorithms, including Deep Neural Network: DNN; K-nearest neighbors: KNN); Support Vector Machine: SVM and Random Forest: (RF). (3) The optimized models were used to predict the independent validation sets. (4) the DNN model was further evaluated by using a “Y-Scrambling”-based permutation test.

Figure 1 provides a schematic overview of the model development procedure performed in this study:

Step 1: we split the 6000 transcriptomic profiles into training (80%), and IV (20%) sets using a stratified splitting strategy, where the same DILI positive/negative (P/N) ratio was kept in both sets. Consequently, we obtained a total of 4800 transcriptomic profiles (P/N ratio = 2854/1946) for the training set and 1200 ones (P/N ratio = 714/486) for the IV set.

Step 2: The MCCV was employed to optimize the model hyperparameters. First, the training set data was stratified split into two subsets with a fixed ratio (i.e., 80% than 20%). Then, the model was developed using 80% of the training set with investigated hyperparameters to predict the remaining 20%. This procedure was repeated 100 iterations. Finally, we used the average predictive results of 100 iterations to select optimized hyperparameters (Result 1).

Based on our previously developed models for DILI prediction, the models tended to provide unbalanced sensitivity and specificity (Chen et al., 2013; Hong et al., 2017). Considering the prevalence of DILI positives and negatives in our dataset, we designed a score function *V* to balance model performance for hyperparameter optimization, as list below:

(4) $$V=\left(\frac{TP^{*}TN-FP^{*}FN}{\sqrt{{\left(TP+FP\right)}^{*}{\left(TP+FN\right)}^{*}{\left(TN+FP\right)}^{*}(TN+FN)}}+00.5\right)*\left(\frac{TN}{TN+FP}\right)$$

Step 3: The model with optimized parameters yielding the highest predictive performance in MCCV was considered as the final model to evaluate the IV set (Result 2).

Step 4: We further evaluate the model by performing a permutation test (i.e., *Y*-scrambling) to investigate whether the predictive performance is better than chance. Specifically, we scrambled the DILI labels of training set 100 times to generate 100 of the pseudo training set. For each pseudo training set, we developed a model based on 80% of data with optimized parameters obtained in Step 2 to predict the remaining 20%. Next, we compared the 100 predictive performances from the pseudo training set with Result 1 from the original training set. Subsequently, we calculated the adjusted *p* value by using Student’s *t*-test. If the adjusted *p* value less than 0.05, we considered the developed model is statistically significant than the random correlation. Moreover, we also used the Cohen’s measure to confirm the findings further.

#### Performance Metrics

We evaluated the predictive performance of developed models by using the area under the receiver operating characteristic (ROC) curve analysis. A ROC curve exhibits the performance of the classification model with a plot by the true predictive rate (TPR) against the false positive rate (FDR). We calculated the area under the ROC curve (AUC) for each developed model. Moreover, three other metrics, including MCC, F1, Cohen’s kappa, accuracy, balanced accuracy, sensitivity, and specificity, were employed to further evaluation of model performance as listed below:

(5) $$\begin{array}{c} \text{MCC}=\frac{T\,P^{*}\,\,T\,N-F\,P^{*}\,\,F\,N}{\sqrt{{\left(T\,P+F\,P\right)}^{*}\,\,{\left(T\,P+F\,N\right)}^{*}\,\,{\left(T\,N+F\,P\right)}^{*}\,\,\left(T\,N+F\,N\right)}} \end{array}$$

(6) $$F\,1=\frac{2\,T\,P}{2\,T\,P+F\,P+F\,N}$$

(7) $$\mathrm{accuracy}=\frac{T\,P+T\,N}{T\,P+T\,N+F\,N+F\,P}$$

(8) $${\mathrm{Cohen}}^{\prime }\,s\,\mathrm{kappa}=\frac{P\,\left(o\right)-P\,\left(e\right)}{1-P\,\left(e\right)}$$

(9) $$\mathrm{balanced}\,\mathrm{accuracy}=\frac{\text{sensitivity}+\mathrm{specificity}}{2}$$

(10) $$\text{sensitivity}=\frac{T\,P}{T\,P+F\,N}$$

(11) $$\text{specificity}=\frac{T\,N}{T\,N+F\,P}$$

Where TP, TN, FP, FN denotes true positive, true negative, false positive, and false negative, respectively. P(o) is the probability of oberserved agreement. P(e) is the probability of random agreement.

### Model Interpretation

#### Applicability Domain

Since drugs in the DILIst dataset covers a wide spectrum of different therapeutic categories, a defined applicability domain is helpful further to apply the developed DNN model in the real-world application. Therefore, we investigated the performance of developed DNN model on each therapeutic category using the WHO Anatomical Therapeutic Chemical (ATC) Classification System (Skrbo et al., 2004). The WHO ATC Classification System is a hierarchical ontology with five different levels. In the study, the first level of ATC was employed, which represents 14 different main therapeutic categories the drug acts on. We first mapped the drugs in the IV set (1200 transcriptomic profiles) onto the first level of ATC. Finally, we calculated performance metrics, including AUC, sensitivity, specificity, and balanced accuracy for each therapeutic category.

#### Functional Analysis

To determine the association between the predictive performance of the developed DNN model and its biological relevance, we performed the Ingenuity Pathway Analysis (IPA), network analysis, and Gene Ontology analysis. We collected the signatures (i.e., top 100 up/down-regulated genes) from each correctly predicted transcriptomic profiles in the IV set. Then, we extracted the top 200 high-frequent genes amongst these signatures. For IPA, we uploaded the 200 high-frequent genes into the IPA web server^2^ to enrich canonical pathways and hepatotoxicity-related functions. For the Gene Ontology analysis, we uploaded the 200 high-frequent genes into the DAVID web server^3^ to enrich GO terms with Benjamini-Hochberg adjusted *p* values less than 0.05. For network analysis, we used the following steps: (1) We queried the STRING version 11 (i.e., a Protein–Protein Interaction (PPI) Networks Functional Enrichment Analysis database) to determine PPIs amongst the 200 high-frequent genes (Szklarczyk et al., 2018); (2) We kept PPIs with confidence scores > 0.7 were considered; (3) We employed the MCODE plug-in for Cytoscape version 3.7.1 to extract and visualize the PPI sub-networks that are densely connected to represent the biological complexes (Bader and Hogue, 2003; Shannon et al., 2003). We set the default parameters in MCODE with Node Score Threshold = ?0.2, *K*-core Threshold = ?2, and MaxDepth = ?100; (4) the genes involved in the enriched subnetworks were input to the IPA software to enrich the pathways and hepatotoxicity-related functions.

### Model Robustness

To further investigate the robustness of the proposed DNN model, we rebuilt the DNN models based on two different strategies, including balanced sampling and drug-based data splitting.

#### Balanced Sampling

Considering the unbalanced DILI positive and DILI negative transcriptomic profiles, we employed a downsampling strategy to rebuilt the DNN models. Specifically, there are 6000 transcriptomic profiles (DILI positive/DILI negative = 3568/2432) used in this study. First, We randomly selected 2432 of 3568 DILI positive transcriptomic profiles with the same number of negative ones. Then, the 4864 (DILI positive/DILI negative = 2432/2432) transcriptomic profiles dataset was stratified split into 80% (3891) for training and 20% (973) for the IV set. Finally, the model was developed by using the training set and test on the IV set. The whole process was repeated 50 times. The average and standard deviation of model performance metrics were calculated.

#### Drug-Based Splitting

Considering the developed DNN model was based on the transcriptomic profiles, the same drug’s transcriptomic profiles may have a chance to present in the training and IV sets. To avoid the potential information leaking occurs, we split the training and the IV sets by drugs to guarantee no common drugs between the two datasets. The total 640 drugs were stratified split into 80% (342 DILI positive/170 DILI negative) for the training set and 20% (85 DILI positive/43 DILI negative) for the IV set. Consequently, the corresponding drug transcriptomic profiles in the training set was used to develop a DNN model. The developed DNN model was evaluated on the corresponding drug transcriptomic profiles in the IV set. The whole process of data splitting and model development was repeated 50 times. The average and standard deviation of model performance metrics were calculated.

### Code Availability

All the models were built using the open-source Python (version 3.6.5). The DNN model was developed with the Keras library version 2.0 on top of TensorFlow version 1.14 as the backend. The models based on conventional ML algorithms (KNN, SVM, and RF) were developed using scikit-learn package version 0.22 (Pedregosa et al., 2011). The model scripts and processed data in this study are available at https://github.com/TingLi2016/L1000_DILI.

## Results

### Model Development

To obtain the optimized model, we comprehensively evaluated model performance under different hyperparameter combinations by using the proposed *V* score function (see “Materials and Methods” section). For DNN, a total 16 of hyperparameter combinations of four activation functions (i.e., Tanh, ReLU, SELU, ELU) and four optimizers (Adam, Adadelta, RMSProp, and SGD) and were investigated. For KNN, we tested the number of neighbors *k* from 3 to 11 with an increased step 2. For SVM, We used a grid search on a polynomial function, and RBF kernel functions with regularization parameter *C* and kernel coefficient gamma γ. The *C* values of 0.01, 0.1, 1, 10, 100 and the γ values of 0.1, 0.01, 0.001, 0.0001 were tested. For RF, We investigated hyperparameter combination with *n_estimators*, *max_depth*, *min_samples_split*, and *min_samples_leaf*. The *n_estimators* was tested from 100 to 500 increased by 100. The *max_depth* of 8, 10, 12, and *None* were employed None means no depth was specified. The *min_samples_split* (i.e., 2, 5, and 10) and *the min_samples_leaf* (1, 2, and 4) were used.

**FIGURE 2:**
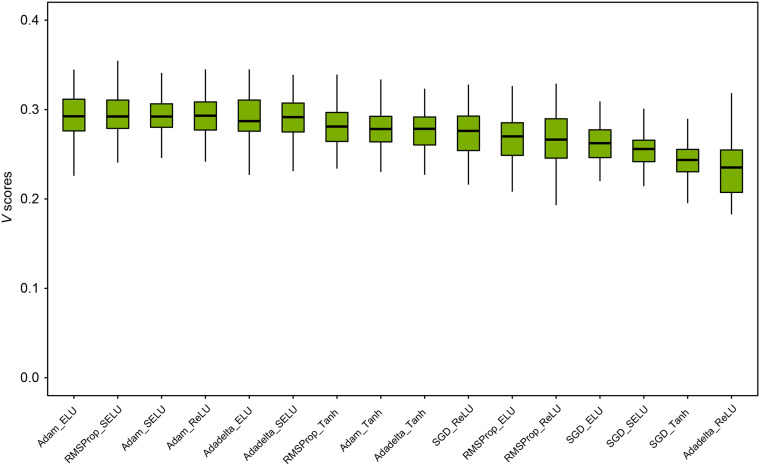
Hyperparameters optimization of DNN models: we investigated a total of 16 hyperparameter combinations of four activation functions (i.e., Tanh, ReLU, SELU, and ELU) and four optimizers (i.e., Adam, Adadelta, RMSProp, and SGD). Each hyperparameter combination was evaluated with a 100-iteration MCCV. The mean and standard deviation of *V* scores is illustrated for each hyperparameter combination. The hyperparameter combination of Elu and Adam was chosen as the best hyperparameters for developing an optimized DNN model.

Figure 2 depicted the distribution of *V* scores of DNN models from the 100-iteration MCCV. The average and standard deviation of *V* scores under 16 hyperparameter combinations were ranked. We observed the *V* score distributions of the top five hyperparameter combinations were no statistically significant difference. We chose the hyperparameter combination with activation ELU and optimizer Adam since they yield the highest average *V* score of 0.295. The detailed *V* score distributions of three conventional classifiers, including KNN, SVM, and RF, were listed in the Supplementary Table S2. For KNN, the optimized number of neighbors *K* was 3. For SVM, the optimized hyperparameter combination of kernel, *C*, and γ was the RBF kernel, 0.0001, and 10, respectively. For RF, the best hyperparameter combination of *estimators*, *max depth*, *min_samples_split*, and *min_samples_leaf* was 500, *None*, 2, and 1, respectively. The average and standard deviation of V scores for the four classifiers were ranked in the following order: DNN (0.295 ± 0.023) > SVM (0.256 ± 0.023) > RF (0.237 ± 0.022) > KNN (0.224 ± 0.025).

**FIGURE 3:**
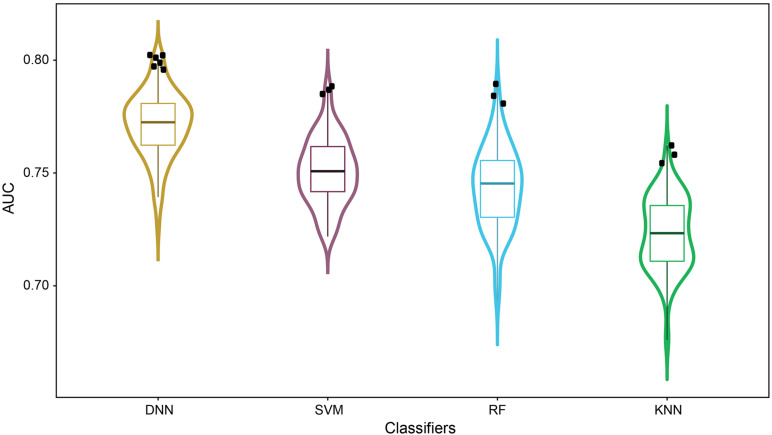
Performance comparison based on MCCV results: the distributions of AUC scores were plotted based on 100-iteration MCCV results of DNN, SVM, RF and KNN in a violin box style. DNN models distributed around 1.5× interquartile range of violin plot were highlighted in black dots.

Figure 3 illustrated the distribution of AUC values from 100-iteration MCCV of the four classifiers. Dots in the violin plot denoted the 100 individual models generated in MCCV. The average and standard deviation of AUC values were decreased in the order DNN (0.772 ± 0.015) > SVM (0.751 ± 0.015) > RF (0.743 ± 0.018) > KNN (0.723 ± 0.016), highlighting the better generalization ability of DNN. Furthermore, more DNN models were distributed around 1.5× interquartile range of violin plot (dots with black color), indicating the higher probability of DNN generating models with better performance. As a result, we selected the model with the highest AUC value for each classifier as the final model.

### DNN Model Provided More Balanced Model Performance

Table 1 listed the performance metrics (i.e., AUC, MCC, F1, Cohen’s kappa, accuracy, balanced accuracy, sensitivity, and specificity) in the training set and IV set across the four classifiers. DNN yielded the highest AUC values (i.e., 0.802 for the training set, and 0.798 for the IV set), Cohen’s kappa (i.e., 0.493 for the training set, and 0.453 for the IV set), and balanced accuracies (i.e., 0.741 for the training set, and 0.721 for IV set) among the four classifiers. Moreover, DNN provided more balanced sensitivity (i.e., 0.851 and 0.839 for the training and IV sets, respectively) and specificity (i.e., 0.630 and 0.603 for the training and IV sets, respectively) than the other three classifiers. RF classifier yielded the highest sensitivity and the lowest specificity in both training and IV sets among the four classifiers, indicating the model suffered a high false-positive rate and tended to predict transcriptomic profiles as DILI positives.

**TABLE 1:** Performance for four different classifiers with optimized hyperparameters.

Dataset	Classifiers	AUC	MCC	F1	Cohen’s kappa	Balanced Accuracy	Accuracy	Sensitivity	Specificity
Training	DNN	**0.802**	0.497	0.809	**0.493**	**0.741**	0.761	0.851	0.630
	KNN	0.762	0.441	0.789	0.436	0.713	0.735	0.834	0.591
	SVM	0.778	0.478	0.805	0.472	0.729	0.753	0.856	0.602
	RF	0.771	0.549	0.837	0.491	0.727	0.774	0.977	0.476
IV	DNN	**0.798**	0.458	0.795	**0.453**	**0.721**	0.743	0.839	0.603
	KNN	0.764	0.409	0.778	0.405	0.698	0.721	0.821	0.574
	SVM	0.777	0.455	0.804	0.438	0.709	0.743	0.888	0.529
	RF	0.747	0.502	0.824	0.436	0.700	0.752	0.975	0.424

*The best performance values among the classifiers.*

**FIGURE 4:**
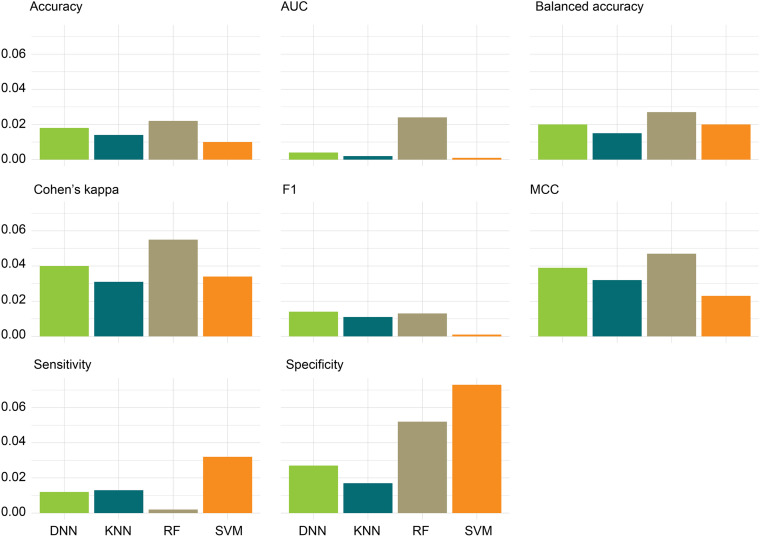
Absolute differences, | MCCV-IV|, of the four performance metrics: absolute differences of the eight performance metrics between the 100-iteration MCCV and the independent validation (IV) set were calculated for the four classifiers.

We further compared the absolute difference of performance metrics between MCCV and IV, denoted as | MCCV-IV| (Figure 4). A large | MCCV-IV| value indicates either potentially overfitting (MCCV > IV) or an unreliable extrapolation (IV > MCCV) of a model. We found that low | MCCV-IV| values (<0.03) of AUC, F1, balanced accuracy, accuracy, and sensitivity for all the four classifiers. RF and SVM had a relatively higher | MCCV-IV| values of specificity (i.e., 0.052 and 0.073) than that of DNN and KNN. RF had a relatively higher | MCCV-IV| values of MCC (0.047) and Cohen’s kappa (0.055) than that of DNN, KNN, and SVM. In general, we did not observe obvious overfitting or underfitting phenomena among the four classifiers. Thus, in the following analysis, we only focused on the DNN classifier because of its superior performance to other classifiers.

### DNN Model Is Significantly Better Than Random Chance

**FIGURE 5:**
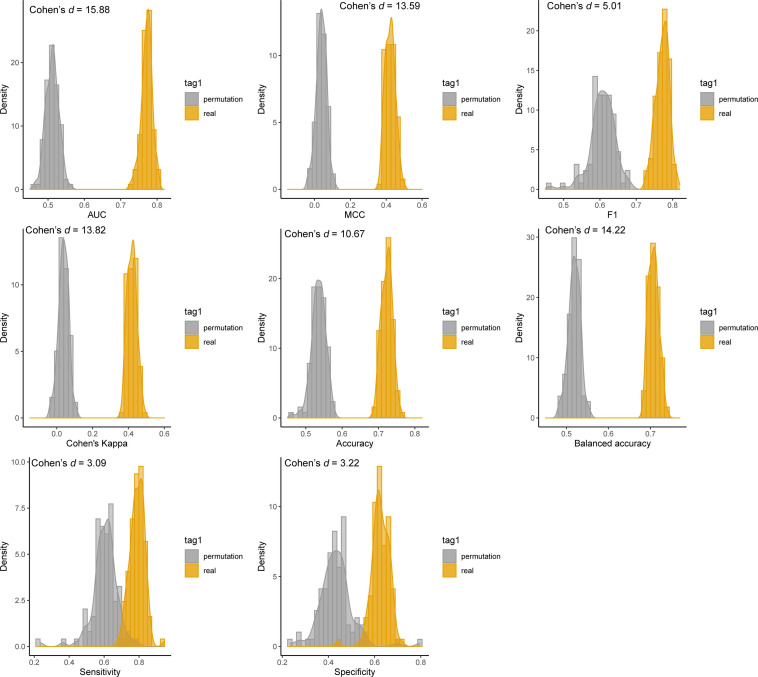
Permutation tests for the developed DNN model: the distributions of the eight performance metrics from 100-iteration MCCV were illustrated. The distributions of DNN models using the real training datasets and *Y*-scrambled training datasets were denoted with orange and gray colors, respectively.

We further investigated whether the predictivity of the developed DNN models yielded by chance. Figure 5 depicted the results of permutation tests for the four performance metrics, including AUC, sensitivity, specificity, and balanced accuracy. In the permutation test, we compared the 1000-iteration MCCV results between the real data and permutated data. The average performance metrics of AUC, MCC, F1, Cohen’s kappa, accuracy, balanced accuracy sensitivity, and specificity (0.772, 0.422, 0.772, 0.419, 0.723, 0.707, 0.790, and 0.625, respectively) derived from real data were much larger than that of permutated data (i.e., 0.512, 0.038, 0.604, 0.038, 0.535, 0.519, 0.603, and 0.434, respectively). The *p* values of eight performance metrics were all less than 2.2E-16, indicting the results yielded from real data are significantly better than random chance. Moreover, we employed the Cohen’s *d* measures to verify further the mean values generated based on real data is statistically different from permutated data. Generally, Cohen’s *d* values more than 0.8 were considered as “large effect” between two distributions. The Cohen’s *d* values were 15.88, 13.59, 5.01, 13.82, 10.47, 14.20, 3.09, and 3.22 for AUC, MCC, F1, Cohen’s kappa, accuracy, balanced accuracy, sensitivity, and specificity, respectively. The large Cohen’s values further demonstrated the predictivity of the developed DNN model is much better than random chance.

### DNN Model Yielded a Better Performance for Oncology Drugs

**FIGURE 6:**
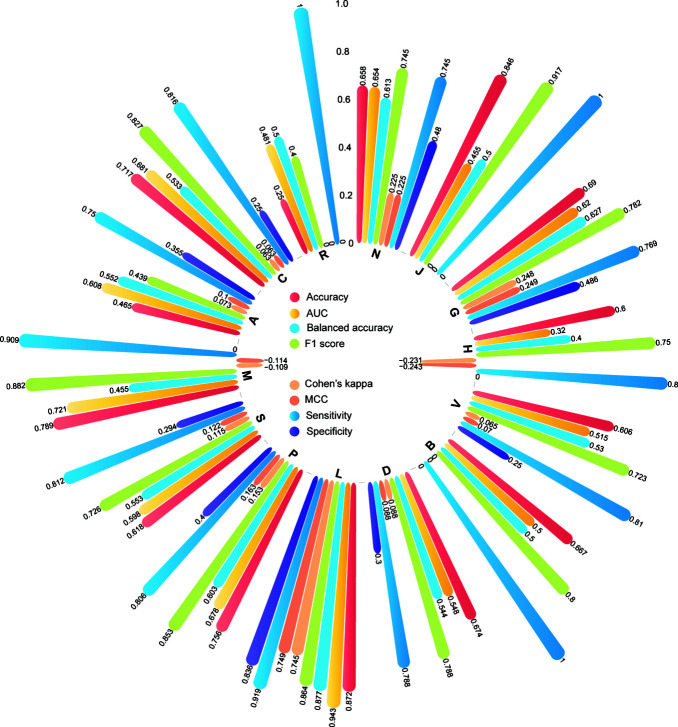
The model performance for the individual therapeutic class according to the first level of the WHO ATC codes.

To define the applicability domain of our developed DNN model, we calculated the performance metrics separately for each therapeutic class in the IV set. Figure 6 showed the distribution of the four performance metrics, including AUC, MCC, F1, Cohen’s kappa, accuracy, balanced accuracy, sensitivity, and specificity for each of 14 ATC therapeutic classes. The predictive power in each therapeutic class varied. *L- antineoplastic and immunomodulating agents* outperformed other therapeutic classes. The higher AUC, MCC, F1, Cohen’s kappa, accuracy, balanced accuracy, sensitivity, and specificity of 0.943, 0.749, 0.864, 0.744, 0.872, 0.836, 0.877, and 0.919 were obtained. We also observed the developed DNN model yielded lower predictive performances (AUC < 0.5) in some therapeutic categories such as *V- various*, *B - blood and blood forming organs*, and *D- Dermatologicals*.

### DNN Model Captured Critical DILI-Related Pathways

**FIGURE 7:**
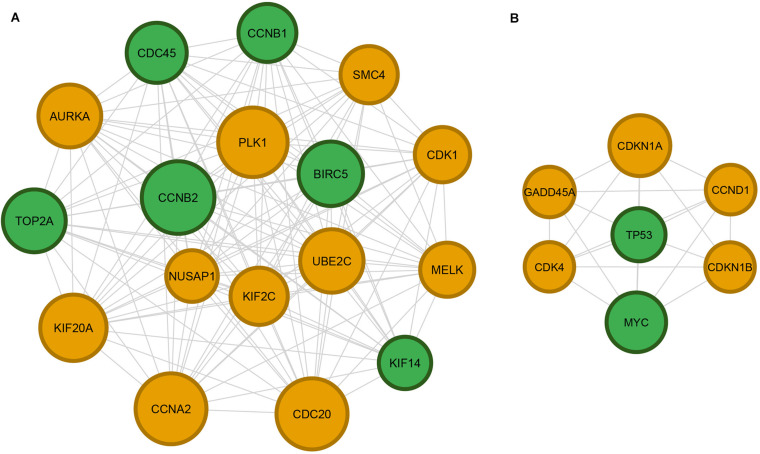
Cytoscape network analysis of protein–protein interactions (PPIs): 482 high confidence PPIs were extracted from the STRING database version 11.0 based on 200 high-frequent genes derived from the DNN model. Panels **(A,B)** are the top 2 subnetworks of the PPI network obtained by using MCODE plug-in for Cytoscape. The hepatoxicity-related genes were highlighted in green color. The size of code is projected based on frequency of genes.

Table 2 listed enriched canonical pathways and hepatoxicity-related functions by using IPA. We used the 200 high-frequent genes of correctly precited transcriptomic profiles in the IV set (see Supplementary Table S3). A total six canonical pathways consisting of *GADD45 Signaling*, *Cell Cycle: G2/M DNA Damage Checkpoint Regulation*, *Estrogen-mediated S-phase Entry*, *Cyclins and Cell Cycle Regulation*, and *ATM Signaling* were enriched with an adjusted *p* value less than 0.05 (see Supplementary Figure S3). Moreover, two hepatoxicity-related functions, including *liver cancer* and *Hepatoblastoma*, were also enriched. Similar findings also were revealed from network analysis (Figure 7), where two high densely interacted subnetworks were extracted by using the MCODE Cytoscape plug-in. We found 6 (*CCNB1*, *KIF14*, *CCNB2*, *TOP2A*, *BIRC5*, and *CDC45*) of a total of 17 genes (35.2%) in the subnetwork one, and two (*TP53*, *MYC*) of seven genes (28.6%) in subnetwork two overlapping with hepatotoxicity-related functions obtained from the IPA (highlighted in green color in Figure 7). A total of eight GO terms were enriched with an adjusted *p* value less than 0.05. Seven of the eight GO terms belonged to the Cellular Component (CC) level, such as the nucleus, cytoplasm, nucleoplasm. The other enriched GO term, positive regulation of apoptotic process, was from the Biological Process (BP) level.

**TABLE 2:** IPA based on 200 high-frequent genes of correctly predicted transcriptomic profiles.

IPA modules	# Genes	Gene Name	*p*-value
**Canonical Pathways**			

GADD45 Signaling	10	CCNB1, CCND1, CCND3, CDK1, CDK4, CDKN1A, GADD45A, GADD45B, PCNA, TP53	2.13E-14
Cell Cycle: G2/M DNA Damage Checkpoint Regulation	9	CCNB1, CCNB2, CDK1, CDKN1A, GADD45A, PLK1, RPS6KA1, TOP2A, TP53	8.66E-09
Estrogen-mediated S-phase Entry	7	CCND1, CDC25A, CDK1, CDK4, CDKN1A, CDKN1B, MYC	1.16E-08
Cyclins and Cell Cycle Regulation	10	CCNB1, CCNB2, CCND1, CCND3, CDC25A, CDK1, CDK4, CDKN1A, CDKN1B, TP53	2.12E-08
ATM Signaling	10	ATF1, CCNB1, CCNB2, CDC25A, CDK1, CDKN1A, GADD45A, GADD45B, NFKBIA, TP53	2.94E-08

**Hepatoxicity-related Functions**			

Liver Cancer	46	ATF5, BIRC5, C2CD5, CANT1, CCDC92, CCNB1, CCNB2, CCND3, CDC45, CGRRF1, CNDP2, CYTH1, DDIT4, DUSP4, GRB10, HMGCS1, HMOX1, HSPA8, HSPD1, IFRD2, IGFBP3, INPP1, INSIG1, JADE2, KEAP1, KIF14, LBR, LGMN, LOXL1, LSM5, MYC, NPC1, NR2F6, NRIP1, POLR2I, RELB, SPR, STXBP1, TIAM1, TIPARP, TLE1, TOP2A, TP53, USP1, WASHC4, WDTC1	2.26E-01
Hepatoblastoma	1	TP53	3.06E-02

**Gene Ontology Analysis**			

Nucleus	55	TOP2A, KDM5B, CDKN1B, FHL2, GABPB1, PRSS23, HOXA10, RGS2, ZFP36, CCND3, TUBB6, CCND1, MYC, RPS6KA1, IER3, TSC22D3, IGFBP3, NOSIP, CDC25A, UGDH, NPEPL1, MELK, BIRC5, ATF5, TP53, RBKS, NOTCH1, PCNA, CDCA4, GLRX, ADRB2, FOXO4, RELB, DNAJB1, CCNB2, CCNB1, NFIL3, USP1, HMOX1, SCAND1, WDTC1, SPDEF, HSPA8, XBP1, SPAG7, GADD45B, UBE2C, TIPARP, SORBS3, PSMB8, NR2F6, FOSL1, NFKBIA, NET1, POLE2	1.96E-03
Nucleolus	20	TOP2A, TCERG1, TLE1, MYO10, PARP2, PLK1, PWP1, ACAT2, JMJD6, CDK4, MYC, NRIP1, RRS1, NUSAP1, TIMELESS, CCDC86, RBM34, POLR2I, RAE1, TP53	6.81E-03
Nucleoplasm	33	TOP2A, ATF1, RNH1, PCNA, STXBP1, KEAP1, FOXO4, TSEN2, SMC4, CNDP2, GNAI2, RELB, CDC20, CCND1, PUF60, SPR, MYC, EPB41L2, HMG20B, CBR3, PARP2, GADD45A, CGRRF1, CDC25B, JMJD6, CCNA2, UGDH, PLCB3, TNIP1, STUB1, ATF5, DLD, RAD9A	1.01E-02
Midbody	7	PLK1, CDK1, KIF14, KEAP1, BIRC5, KIF20A, GNAI2	2.27E-02
Cytoplasm	52	KDM5B, RNH1, CASC3, TSEN2, SMC4, CNDP2, HOXA10, CDC20, PCMT1, RGS2, CCND3, TUBB6, PNP, CCND1, SPR, KIF5C, RPS6KA1, NUSAP1, PARP2, TSC22D3, NOSIP, KCTD5, CDC25A, CDC25B, NPEPL1, MELK, BIRC5, ATF5, TP53, RBKS, PCNA, INPP1, PRUNE1, HSPD1, GNAI2, EPB41L2, CYTH1, UBQLN2, MYO10, GADD45B, UBE2C, GADD45A, PLK1, SORBS3, PSMB8, MLLT11, EIF5, GNPDA1, TNIP1, BAMBI, CDK1, RAD9A	2.27E-02
Spindle Microtubule	5	PLK1, NUSAP1, CDK1, BIRC5, AURKA	2.27E-02
Cyclin-Dependent Protein Kinase Holoenzyme Complex	4	CCND3, CDKN1A, CCND1, CDK4	3.36E-02
Positive Regulation Of Apoptotic Process	10	MLLT11, TOP2A, NET1, NOTCH1, MELK, GADD45B, GADD45A, IGFBP3, HMOX1, SPDEF	2.75E-02

### DNN Provided Robust Models

To further investigate the proposed DNN model’s robustness, we redeveloped the models based on two sampling strategies, including balanced sampling and drug-based splitting.

**FIGURE 8:**
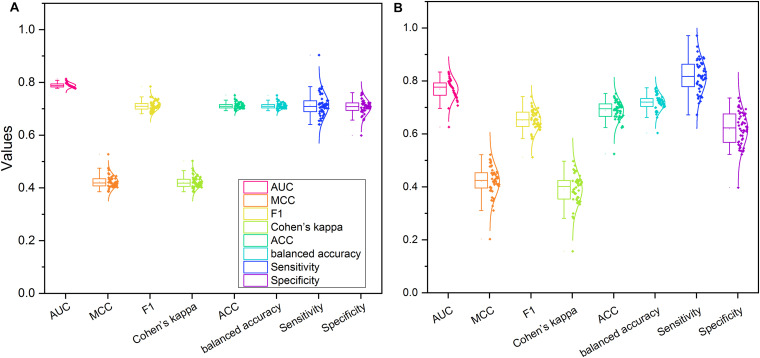
The performance of the proposed DNN model based on the two different sampling strategies: **(A)** balanced data sampling; **(B)** drug-based data splitting.

Figure 8A illustrated the distribution of the eight performance metrics of 50 IV sets based on the balanced sampling strategy. The average and standard deviation of AUC, MCC, F1, Cohen’s kappa, accuracy, and balanced accuracy were 0.789 ± 0.009, 0.423 ± 0.025, 0.711 ± 0.019, 0.422 ± 0.023, 0.711 ± 0.011, and 0.711 ± 0.011, respectively. As expected, the balanced sampling method provided a more balanced sensitivity (0.714 ± 0.045) and specificity (0.707 ± 0.029) compared to the original dataset (see Supplementary Table S4). Furthermore, we observed that the average AUC (0.789) yielded based on balanced sampling was slightly lower than the AUC (0.798) generated with the original dataset, indicating the unbalanced positive and negative sample did not influence the performance of the developed DNN model.

Figure 8B showed the performance metrics of 50 IV sets using the drug-based splitting strategy. The average and standard deviation of AUC, MCC, F1, Cohen’s kappa, accuracy, balanced accuracy, sensitivity, and specificity were 0.769 ± 0.038, 0.420 ± 0.057, 0.651 ± 0.041, 0.391 ± 0.059, 0.689 ± 0.038, 0.718 ± 0.030, 0.818 ± 0.061, and 0.619 ± 0.066, respectively (see Supplementary Table S5). We found that 11 of 50 IV datasets with drug-based splitting methods had a better AUC value (i.e., AUC ≥ 0.798), suggesting no obvious information leaking exists in the DNN model developed with the original datasets.

## Discussion

Drug-induced liver injury remains one of the largest safety concerns in the drug development characterized by the complicated intrinsic and idiosyncratic mechanisms (Andrade et al., 2019). Although significant efforts have been made to improve DILI prediction ability, the performances of reported models are still suboptimal. Thus, in this study, we developed an 8-layer DNN model to investigate how to take advantage of accumulated transcriptomic profiles to enhance DILI prediction. It is the first attempt to use the largest binary DILI classification data -DILIst and transcriptomic profiles from the LINCS L1000 dataset to develop the DNN model. The developed DNN model yielded the best predictive results among the four different ML classifiers with AUCs of 0.802 and 0.798 and balanced accuracies of 0.741 and 0.721 for the training and an IV set, respectively. Furthermore, Our DNN model provided more balanced sensitivity and specificity (Table 1).

A defined applicability domain of the developed model is of help to its real-world application. The therapeutic class analysis demonstrated that the developed DNN model was specifically effective (e.g., 0.943 AUC) in predicting DILI potential of *L- antineoplastic and immunomodulating agents*. It would be valuable to improve patient quality of life during drug treatment by reducing the potential for hepatotoxicity in this class of drugs. However, the diversity in chemical class within the antineoplastic and immunomodulating agents, together with the underlying complex biology, is a challenge for the identification of the potential for clinical hepatotoxicity. The model described here has great potential to overcome this during the early stages of drug development as soon as efficacy data generated in cancer cell lines become available. Once in clinical development, the potential for hepatotoxicity is routinely monitored alongside other parameters to ensure patient safety.

An explainable *in silico* models is a pre-requisite for a better understanding of the underlying mechanism and causal relationship between data and biological endpoints. Through the functional analysis, we found five canonical pathways and two hepatotoxicity-related functions by using IPA based on 200 high-frequent genes derived from the DNN model. The results were also confirmed by using the network analysis. Several canonical pathways have been reported to play essential roles in DILI pathogenesis and etiology. For example, it was reported ATM signaling pathway plays an important protective function of causing liver failure in mice (Bandi et al., 2011). Furthermore, it was reported that Estrogen receptor alpha (ESR1)-mediated signaling inhibits liver regeneration by downregulation of Wnt signaling resulting in lower cyclin D1 activation in ESR1 knockout rats (McGreal et al., 2017).

It is worthwhile to consider some additional studies to confirm our findings in this study and further verify our developed DNN in the real-world application. First, we did not investigate the model performances based on transcriptomic profiles generated from different genomics platform. Based on our previous study, the extrapolation ability between assay platforms are multiple-factorial, and a careful selection of cell lines is strongly recommended (Liu et al., 2017, 2018, 2020). However, considering the ongoing declining cost of the emerging genomics technology such as L1000 technologies, it is affordable for most of the researches to screen the investigated drug candidates. Second, DL is growing at a very rapid rate. In the current study, we just employed the DNN algorithm for a proof-of-concept purpose. Other DL algorithms, such as transfer learning (Pan and Yang, 2009) and multi-task learning (Ruder, 2017), were worth investigating for further improving DILI perdition. Finally, in our current study, we only fine-tune the essential parameters, including activation functions and optimizers in the DNN model. Other parameters, such as the number of hidden layers and node numbers, were arbitrarily selected. With that said, the prediction performance of the developed model may be further improved by extra fine-tuning.

Drug-induced liver injury is a multifactorial endpoint that cannot easily be predicted by current pre-clinical animal models. The results presented illustrate the utility of a DL algorithm combined with transcriptomics information for predicting DILI. The developed DNN model could be a promising tool for DILI potential detection in the early stages of drug development.

## Data Availability Statement

The datasets presented in this study can be found in online repositories. The names of the repository/repositories and accession number(s) can be found in the article/Supplementary Material.

## Author Contributions

ST, ZL, and WT conceived and designed the study. TL and ZL performed the data analysis. TL, ZL, and ST wrote the manuscript. RR, ST, ZL, and WT revised the manuscript. All authors read and approved the final manuscript.

## Disclaimer

The views presented in this article do not necessarily reflect current or future opinion or policy of the U.S. Food and Drug Administration. Any mention of commercial products is for clarification and not intended as an endorsement.

## Conflict of Interest

RR is co-founder and co-director of ApconiX, an integrated toxicology and ion channel company that provides expert advice on non-clinical aspects of drug discovery and drug development to academia, industry, and not-for-profit organizations. The remaining authors declare that the research was conducted in the absence of any commercial or financial relationships that could be construed as a potential conflict of interest.

## Footnotes

1 https://pubchem.ncbi.nlm.nih.gov/idexchange/idexchange.cgi

2 https://apps.ingenuity.com/ingsso/login

3 https://david.ncifcrf.gov/

## Supplementary Material

Supplementary Figure 1 The distrubition of Euclidean distances among the 23,791 transcriptomic profiles of LINCS L1000 data.

Supplementary Figure 2 The model structure of the developed DNN model.

Supplementary Figure 3 Enriched canonical pathways by using IPA.
