# Supplementary figures and images for: Deep Learning on High-Throughput Transcriptomics to Predict Drug-Induced Liver Injury
Source: Front Bioeng Biotechnol. 2020 Nov 27;8:562677. doi: 10.3389/fbioe.2020.562677 (PMC7728858; doi:10.3389/fbioe.2020.562677)

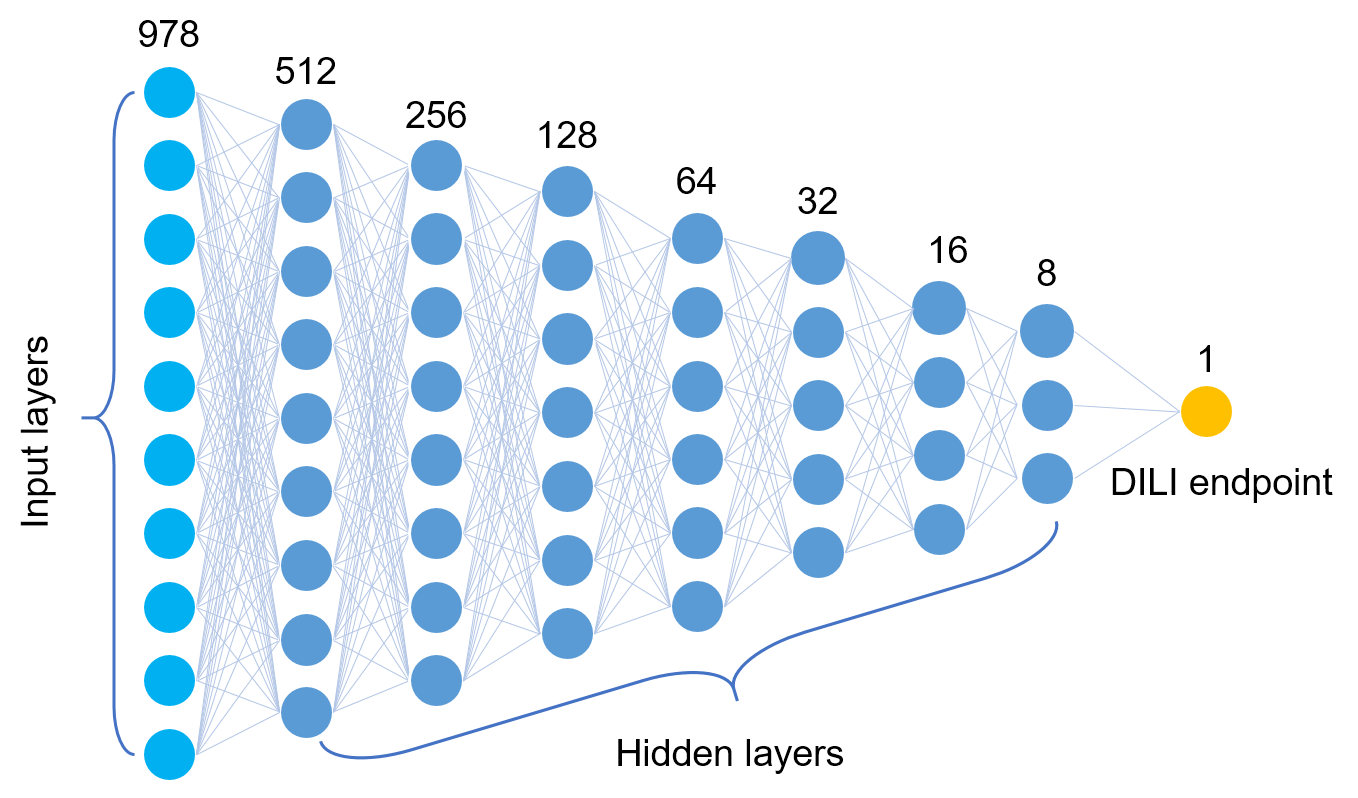

Supplement: Supplementary Figure 2 — The model structure of the developed DNN model. [file Image_2.tif]

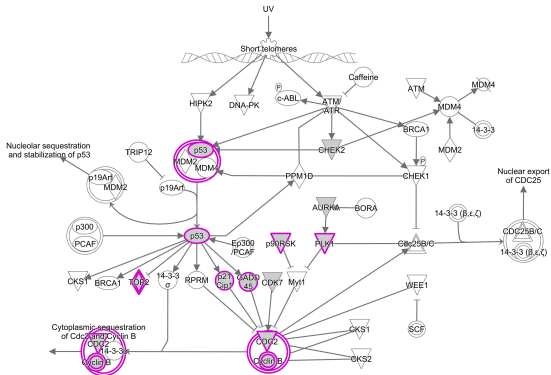





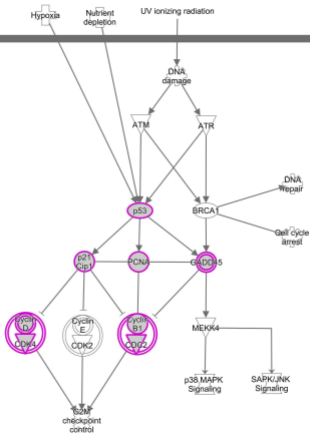

Supplement: Supplementary Figure 3 — Enriched canonical pathways by using IPA. [file Image_3.pdf]
